# Animal Chlamydiae: A Concern for Human and Veterinary Medicine

**DOI:** 10.3390/pathogens11030364

**Published:** 2022-03-17

**Authors:** Hanna Marti, Martina Jelocnik

**Affiliations:** 1 Institute of Veterinary Pathology, Vetsuisse-Faculty University of Zurich, 8057 Zurich, Switzerland; 2 Centre for Bioinnovation, University of the Sunshine Coast, Sippy Downs 4556, Australia

The Chlamydiae are a phylum of obligate intracellular, Gram-negative bacteria with a biphasic lifecycle. Commonly found in the environment, they infect a variety of hosts, including amoebae, insects, aquatic animals, reptiles, birds, and mammals (including humans), with *Chlamydiaceae* representing the most important pathogenic family in the phylum [1,2]. Apart from the strict human pathogen *Chlamydia (C.) trachomatis*, the successful and enigmatic animal-associated species remain in the spotlight as significant pathogens of wildlife; domesticated animals; pets; and potentially, humans. *C. suis, C. abortus, C. pecorum*, and *C. psittaci* infect a wide range of livestock hosts (pigs, sheep, cattle, horses, and poultry) and may cause diseases resulting in economic losses [1]. 

However, these same species also readily infect wildlife hosts. The best examples are *C. pecorum*, a species globally known as the ‘koala chlamydia’, causing severe ocular and urogenital infections in koalas, and *C. psittaci*, a species infecting a wide range of birds as well as unusual hosts, such as wallabies, rabbits, and guinea pigs. Other species such as *C. felis* and *C. caviae* are typically restricted to domestic pet hosts, cats, and guinea pigs; however they also pose a risk to humans, specifically pet owners [1]. 

Despite considerable progress in recent years concerning the diagnostic identification as well as our molecular and cell biological understanding of these different species, many questions remain regarding their genetic diversity, epidemiology, and pathogenesis. Accordingly, we assembled a collection of original research articles, brief reports, and a review from researchers across Asia, Australia, Europe, and North America to advance our knowledge of these veterinary *Chlamydiaceae* species and to clarify their role as pathogens in both veterinary and human medicine.

## 1. Chlamydial Infections in Livestock: A Global Concern for Economic and Production Losses

*Chlamydia suis* may cause conjunctivitis, enteritis, pneumonia, pericarditis, and polyarthritis in piglets and reproductive problems in sows [3]. Furthermore, *C. suis* is the only chlamydial species known to have naturally acquired a *tetA*(C)-encoded efflux pump conferring resistance to tetracycline [4]. 

*C. suis* was the focus of a study in Alabama, USA, comparing the prevalence in domestic pigs to that of wild boars. With peptide ELISA antibody prevalence of 13.0% and 80.0% for feral swine and domestic pigs, respectively, *C. suis* is much more common in domestic pigs, possibly promoted by crowding. These results were confirmed by comparative FRET-qPCR [5]. 

While *C. suis* is associated with conjunctivitis or keratoconjunctivitis in pigs [3,6], our understanding of *C. suis* ocular infections is still limited. Unterweger and colleagues present the first in vivo study, in which an experimental mono-infection with *C. suis* produced clinical signs of conjunctivitis in piglets [7]. Apart from eyes, the authors detected *C. suis* in the lungs and intestines of infected piglets, suggesting systemic infection, but no *C. suis*-specific antibodies were detected over the course of the three-week study period.

With the long-term aim of reducing the use of antibiotics and thus the risk of antibiotic resistance development, De Puysseleyr and colleagues investigated the potential effects of different transferrins—immunmodulatory glycoproteins responsible for iron transport to tissues—on *C. suis.* In a brief report, the authors showed that bovine lactoferrin significantly reduced the inclusion size of *C. suis* in McCoy cells and that this anti-chlamydial activity was also effective in semen samples spiked with *C. suis* [8].

*Chlamydia abortus* causes enzootic abortion of ewes (EAE). This economically important disease affects the global sheep industry, except for in Australia and New Zealand [1]. However, in certain regions, such as Saudi Arabia, information on *C. abortus* infections in sheep and goats remain scarce. Fayez and colleagues analysed 1717 sheep and 1101 goat serum samples to characterise the infection dynamics of *C. abortus* in Eastern Saudi Arabia. The *C. abortus* seroprevalence of 11.1% and 10.6% was found for sheep and goats, respectively. Regarding improved flock management, the authors identified the introduction of new animals into the flock as a risk and good farm hygiene as a protective factor [9]. 

*Chlamydia pecorum* infections are endemic in livestock, with most infections being clinically inapparent and characterised by faecal shedding. However, *C. pecorum* continues to be associated with clinical disease, such as encephalomyelitis, polyarthritis, conjunctivitis, enteritis, mastitis, and reproductive disorders, including sporadic cases of abortion, which could cause substantial losses to the producer. Recently, in Australia, *C. pecorum* has been detected and associated with naturally occurring ovine abortions [10]. The authors found that the seropositivity rate of ewes with lamb loss was higher compared with unaffected animals. Additionally, they provide the first whole genome sequence (WGS) of the Australian abortigenic (sequence type) ST23 strain and found that the strain possesses a cryptic plasmid with a unique deletion in coding sequence 1 (CDS1).

*C. pecorum* detection is often only performed in specialised diagnostic laboratories. To overcome these limitations, loop-mediated isothermal amplification (LAMP) has been proposed as a Point of Care (POC) rapid diagnostic method for koala *C. pecorum* and equine *C. psittaci* infections [11]. To evaluate the use of a *C. pecorum* LAMP assay for sheep infections, Clune and colleagues found that a *C. pecorum* LAMP assay agreed with the reference qPCR results in 80.4% and 85.71% when testing ovine swab and tissue samples, respectively. The authors concluded that, following further optimisation, LAMP testing is promising as a simple, low-cost, and accurate POC *C. pecorum* detection method [12].

Salpingitis leads to economic losses in laying hens and breeder ducks in China. After identifying *Escherichia coli*, *Enterococcus faecalis*, and *C. psittaci* in the oviducts of diseased hens and ducks, Fang and colleagues inoculated laying hens with isolates of these bacteria. These hens developed salpingitis, which was more severe in animals infected with all three bacteria compared with corresponding single infections [13]. Interestingly, in breeder ducks, a single infection with *C. psittaci* but not a single infection with *E.a coli* or *E.s faecalis* had a negative impact on duck oviduct health, and co-infection led to salpingitis.

## 2. Chlamydial Infections in Wildlife: Concerns for Spill-Over at the Interface of Wildlife, Domesticated Animals, and Humans

Chlamydial infections in wildlife are interesting but not surprising, considering (a) anthropogenic and environmental factors, and (b) the active human/pet/livestock/wildlife interface. As such, these infections raise many concerns, including the risks they pose directly to wildlife health and conservation, spillback, and/or spill-over to domesticated animals [14] and potential for zoonotic disease in humans. 

An important example of the above is the koala chlamydial disease, one of the most researched wildlife diseases in Australia. *Chlamydia*-infected wild koalas are treated with antibiotics, but the survival rate of koalas in wildlife hospitals is only around 50%. These high mortality rates are often at least partially caused by reproductive cysts in the female reproductive tract, leading to infertility and potentially euthanasia [15]. Phillips and colleagues noted a considerable knowledge gap regarding the aetiology and pathogenesis of reproductive cysts because the diagnostic work-up is often limited to ultrasound scans. The authors concluded that further investigations are urgently needed to improve treatment of *Chlamydia*-infected koalas and to reduce these high mortality rates [16]. 

The prevalence and role of the *Chlamydiaceae* in sympatric wild and domesticated animals is also of interest due to the potential for cross-host infections [17]. Specifically, a study investigating mountain habitats in northern Spain showed that 0.6% of the free-range Pyrenean chamois (*Rupicapra pyrenaica*) and 1.4% of the domestic sheep, from a total of 893 animals evaluated, were positive for *Chlamydiaceae*, with *C. pecorum* being the only species identified. There was no association between the detection of the *Chlamydiaceae* and infectious keratoconjunctivitis caused by *Mycoplasma conjunctivae* [18]. Nevertheless, further studies are needed to better understand the ecology of *C. pecorum* and its possible role as a ruminant pathogen at the wildlife–livestock interface.

Perhaps the best example of a chlamydial pathogen thriving on the wildlife–livestock–human interface is *C. psittaci*, a chlamydial species infecting over 450 species of birds and infecting humans and livestock (cattle, sheep, and pigs). Additionally, in Australia, infections of pregnant Thoroughbred mares have been reported, leading to abortion in the mare and causing novel zoonotic disease in veterinarians [19,20]. Anstey and co-workers investigated the genetic diversity of *C. psittaci* strains from avian, equine, marsupial, and bovine hosts and found that clonal ST24/*ompA* genotype A strains dominate *C. psittaci* infections in Australia. These ST24/*omp*A genotype A strains pose a documented zoonotic risk to humans. The authors further discovered a novel strain (ST306) in the Western brush wallaby, a novel host of *C. psittaci* [17]. The multidisciplinary approach in this study ranging from equine infectious disease to ecology is in line with the “One Health” perspective [21].

Complementing the study above and further highlighting the need to implement the One Health concept, the report by Chaber and team describes two cases of human psittacosis following exposure during the dissection of an infected Rosella parrot in Australia, which was infected with an ST24/*ompA* genotype A *C. psittaci* strain [22]. One patient was hospitalized with pneumonia, and in both cases, the disease was not diagnosed during routine medical investigations. Epidemiological and clinical evidence were crucial for the final diagnosis, leading the authors to conclude that awareness of the disease as well as communication between veterinary and human health services must be improved.

## 3. Avian Infections: New Chlamydial Species and Hosts, and New Concerns for Avian Health?

Avian chlamydiae are increasingly gaining attention worldwide, as a recent genus expansion has contributed to new species detection in a broad range of avian hosts. In a comprehensive review, Stokes and colleagues present an update on chlamydial infections in wild avian populations [23]. They summarised the increasing global diversity and host range, elaborated on the expected clinical signs in wild birds, and emphasised the risk of zoonotic transmission and its implications for avian conservation.

In a study from Switzerland, 1128 samples from 341 raptors and 253 corvids were analysed [24]. Over 20% of the corvids and almost 6% of the raptors were positive for *Chlamydiaceae,* with *C. psittaci* being the most frequently detected chlamydial species [24]. Using *omp*A genotyping, the most commonly identified *C. psittaci* genotype was 1V, which is often found in corvids, but zoonotic genotype A was also identified. The study found no cases of *C. buteonis* [24], a recently identified species in raptors [25]. The authors concluded that chlamydia-infected raptors and crows may pose a risk of zoonotic infection for those who work with or regularly come in close contact with these birds such as zoo or pet shop workers, pet bird owners, and veterinarians [24]. 

## 4. Chlamydial Infections in Domesticated Pets: Common Pathogens with Zoonotic Potential

Chlamydial infections in domestic pets, such as cats and guinea pigs, have also been in the spotlight due to serious disease in affected animals as well as zoonotic infections in their owners [26]. Bressan and colleagues investigated the occurrence of *Chlamydia felis* in 309 stray and 86 pet cats from Switzerland and found that nearly 20% of stray cats and around 12% of pet cats were positive for *Chlamydiaceae*, most of which were later identified as *C. felis*. However, *C. abortus* was also found in rare cases. Moreover, there was a correlation between chlamydial positivity in the eye and signs of conjunctivitis. While ocular infection was more common, *C. felis* was also found in rectal swabs. Finally, this study confirmed the highly conserved nature of the *C. felis* genome [27].

In a joint study between the Netherlands and Switzerland, Ciuria and colleagues investigated the occurrence of *Chlamydia* in guinea pigs and rabbits [28]. Overall, the chlamydial prevalence was 2.3% and 12.5% for Swiss and Dutch guinea pig samples, respectively. The most commonly identified species was *C. caviae*, with their *ompA* sharing 100% nucleotide identity with the strains that caused severe pneumonia in humans [26,28]. Lastly, while *C. caviae* was not detected in rabbits, the zoonotic *C. psittaci* genotype A was detected in two rabbit and in two guinea pig samples from Switzerland.

## 5. Chlamydial Genomics: A Tool of Choice to Understand Evolution, Diversity, Epidemiology, and Pathogenicity of Chlamydial Veterinary Pathogens

Traditional genotyping techniques, such as *Chlamydiales* MLST and/or other gene markers schemes, continue to prove valuable in surveillance and genetic diversity studies of chlamydial veterinary pathogens. However, genomic investigations are becoming widely used in bacterial studies, as they provide fine-detailed insights into epidemiology, genetic diversity, and evolution as well as genome biology. Using a novel genome analysis tool termed Roary ILP Bacterial Annotation Pipeline (RIBAP), Holzer and colleagues analysed 33 chlamydial strains from 12 species, aiming to identify characteristic features of *C. psittaci*. The authors describe a <30 kilobase pair (kbp) plasticity zone, a set of *C. psittaci*-specific inclusion membrane (Inc) proteins, particularly IncA, B, C, V, X, and Y; an uncommonly large SinC protein; a type III-secreted effector and potential virulence factor; and a total of 14 polymorphic membrane proteins (Pmp) of the subtype G [29]. Some of these elements were also detected in other chlamydial genomes.

To taxonomically classify the recently identified group of avian *C. abortus* strains, Zareba-Marchewka and team used hybrid resequencing to conduct in-depth genome analysis of draft genomes [30]. The authors found that genotypes G1, G2, and 1V, isolated from Eurasian teal, mallards, and magpies, respectively, share a closer relationship with the ruminant *C. abortus* strain and show features typical for the *C. psittaci* species [30]. 

To date, only five draft genomes of *C. pecorum* are available, hampering our understanding of this koala pathogen, its virulence factors, and its diversity among koalas and other host species. White and colleagues produced closed genomes of the two koala *C. pecorum* strains DBDeUG_2018 and MC/MarsBar_2018. They re-evaluated their genomic make-up and discovered new loci of interest that could distinguish between northern and southern koala strains. They also provided new information on putative secreted effectors, which may act as novel virulence factors [31]. These findings establish the foundation for further work toward identifying and understanding host specificity and adaptation of koala chlamydial infections.

## 6. Future Directions for Chlamydial Veterinary Research and Practice

This comprehensive collection about a broad variety of animal Chlamydiae clearly demonstrates the interest of the research community in these fascinating and unique bacteria. Their zoonotic potential as well as their impact on endangered wildlife, economically important livestock, and beloved pets attracts the attention of not only the public but also veterinary and human healthcare workers. A very recent and particularly relevant example is a report of fatal *C. psittaci*-induced pneumonia in a human patient with COVID-like symptoms [32]. Considering the findings in this diverse collection of studies, we propose that future directions in the chlamydial field should encompass the following:

- A One Health approach to chlamydial infections and disease;
- An increased global collaborative effort to understand the diversity of veterinary chlamydial species, including their variation in strain or species virulence as well as their pathogenic and zoonotic potential;
- More widespread use of WGS and genomic analyses;
- The implementation of novel POC diagnostics to more rapidly manage acute chlamydial disease; and
- Vaccine development towards the prevention of chlamydial diseases in livestock, wildlife, and companion animals.

In conclusion, while this collection has answered a number of questions, many challenges remain regarding our continued efforts to better understand these unique and versatile bacteria. However, these are exciting times for chlamydial veterinary research globally, and we cannot wait for the amazing discoveries yet to be made. 
